# In search of induction and latency periods: Space-time interaction accounting for residential mobility, risk factors and covariates

**DOI:** 10.1186/1476-072X-6-35

**Published:** 2007-08-23

**Authors:** Geoffrey M Jacquez, Jaymie Meliker, Andy Kaufmann

**Affiliations:** 1 BioMedware, Ann Arbor, USA; 2 Department of Environmental Health Sciences, The University of Michigan, Ann Arbor, USA

## Abstract

**Background:**

Space-time interaction arises when nearby cases occur at about the same time, and may be attributable to an infectious etiology or from exposures that cause a geographically localized increase in risk. But available techniques for detecting interaction do not account for residential mobility, nor do they evaluate sensitivity to induction and latency periods. This is an important problem for cancer, where latencies of a decade or more occur.

**Methods:**

New case-only clustering techniques are developed that account for residential mobility, latency and induction periods, relevant covariates (such as age) and risk factors (such as smoking). The statistical behavior of the methods is evaluated using simulated data to assess type I error (false positives) and statistical power. These methods are applied to 374 cases from an ongoing study of bladder cancer in 11 counties in southeastern Michigan, and the ability of the methods to localize space-time interaction at the individual-level is demonstrated.

**Results:**

Significant interaction is found for induction periods of ~5 years and latency ~19.5 years. Data are still being collected and the observed clusters may be attributable to differential sampling in the study area.

**Conclusion:**

Residential histories are increasingly available, raising the possibility of routine surveillance in a manner that accounts for individual mobility and that incorporates models of cancer latency and induction. These new techniques provide a mechanism for identifying those geographic locations and times associated with increases in cancer risk *above and beyond *that expected given covariates and risk factors in geographically mobile populations.

## Background

Cluster analysis provides an objective basis for evaluating whether geographic cancer patterns are significant [1,2]. Dozens of approaches are now available (e.g., [3-10]); however, most of these were developed for spatially static datasets and assume individuals are immobile and that latency is negligible [11]. Most published studies still rely only on place of residence at time of diagnosis or of death to record the locations of health events. But when analyzing cancers, causative exposures may occur many years prior to diagnosis, and during this interval individuals may move place of residence. Failure to account for residential mobility, therefore, can make detecting clustering of cases in relation to causative exposures difficult or even impossible. Recent studies demonstrate that results obtained using static spatial point distributions can lead to erroneous conclusions regarding the timing, existence, extent, and locations of disease clusters [12,13]. Tests for space-time interaction that account for residential mobility thus are required when studying cancer.

For cancer, interaction statistics allow researchers to explore two different types of etiological hypotheses: infectious processes (e.g. cancers with viral origins), and geographically and temporally localized exposures to carcinogenic agents (e.g. exposure to radon in home environments). In addition, interaction tests have the substantial advantage of working with cases-only data, and do not require the selection of controls. The development of appropriate interaction tests that account for residential mobility, risk factors, covariates and reasonable models of latency and induction periods is expected to be a significant methodological advance that will allow researchers to work directly with data from cancer registries without the need for the painstaking selection of matched controls.

In 1967 Nathan Mantel [14] proposed a space-time interaction test for case data, and represented the observations as {*x*_*i*_, *y*_*i*_, *t*_*i*_}. Here *x*_*i*_, *y*_*i *_is the place of residence for the *i*^th ^case, and *t*_*i *_is the time of diagnosis or death. "Interaction" arises when nearby cases occur at about the same time, and may indicate a contagious process such as infection transmission, or a geographically and temporally localized exposure to a carcinogen. For infection the underlying assumption is that nearby individuals are more likely to interact and experience infection transmission events. For a localized exposure the assumption is that nearby individuals will experience similar exposures such that their disease risk will be elevated at about the same time.

The proximity metrics underlying Mantel's test are the spatial and temporal distances between pairs of cases. Knox [15] used adjacencies, Diggle et al. [16] the K-function and Jacquez [17] nearest neighbor relationships. Recent adaptations to Knox's method account for changing population size [18] and the time required for infection transmission [19], but do not account for human mobility. In studies of cancer clustering, methods have yet to effectively account for latency, perhaps because latency is difficult to observe, and our knowledge of it is uncertain. This becomes increasingly problematic when we consider residential mobility. The average American now moves every 5–7 years, meaning that at time of diagnosis few cases actually reside where causative exposures may have occurred [20]. And no tests for interaction simultaneously account for human mobility, latency, risk factors and covariates. This paper introduces novel techniques that account for residential mobility, cancer latency, risk factors and covariates, evaluates them using simulations, and then applies them in a study of bladder cancer in southeastern Michigan.

## Methods

We begin with descriptions of the empirical induction period (EIP), notation, models of EIP and metrics for evaluating residential proximity for mobile individuals. We then derive space-time interaction tests that incorporate EIP and residential mobility. Next, we extend these to adjust for risk factors and covariates. We then define the algorithm used to evaluate sensitivity of the interaction statistics to specification of the EIP. Finally, we apply the new methods to (a) simulated data for which the extent of interaction is known and (b) residential histories of bladder cancer cases in Michigan.

Rothman [21] recognized that illness in an individual may have a multiplicity of causes, none of which alone may be sufficient to cause the disease. This makes definition and observation of disease latency problematic. He recommended that one explore sensitivity of latency-based metrics by evaluating a range of plausible empirical induction periods. We define the EIP as an induction period, *ω*, in which causative exposures occurred, and a lag, *τ*, the latency. In practice *ω *and *τ *are unobservable, and we therefore explore sensitivity of interaction to specification of these parameters.

**Figure 1:**
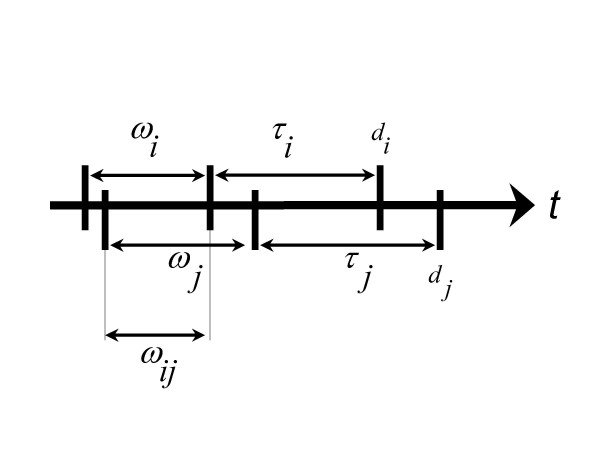
Model of empirical induction periods. The date of diagnosis for the *i*^th ^case is *d*_*i*_. *τ*_*i *_is the temporal lag between initiation of the disease (e.g. appearance of the first cancer cells) and diagnosis. *ω*_*i *_is the induction period when causative exposures occurred. *ω*_*ij *_is that time interval when the induction windows for cases *i *and *j*, *ω*_*i *_and *ω*_*j*_, overlapped.

Let *d*_*i *_represent the time of diagnosis of case *i*. This could be time of death or another event in the life course, but for exposition we use time of diagnosis. The locations where a person resides during *ω *is called the *exposure trace *[12]. We subscript the induction period, *ω*_*i*_, and latency, *τ*_*i*_, so that they can differ across cases. Now consider cases *i *and *j*. Define *ω*_*ij *_as the interval when *ω*_*i *_overlaps *ω*_*j *_(Figure 1, Equation 1).

*ω*_*ij *_= *ω*_*i *_∩ *ω*_*j*_

A measure that accounts for residential mobility and co-occurrence of induction periods is then

ηijkω={1 iff i and j were ever k nearest neighbors during ωij0otherwise.
 MathType@MTEF@5@5@+=feaafiart1ev1aaatCvAUfKttLearuWrP9MDH5MBPbIqV92AaeXatLxBI9gBaebbnrfifHhDYfgasaacH8akY=wiFfYdH8Gipec8Eeeu0xXdbba9frFj0=OqFfea0dXdd9vqai=hGuQ8kuc9pgc9s8qqaq=dirpe0xb9q8qiLsFr0=vr0=vr0dc8meaabaqaciaacaGaaeqabaqabeGadaaakeaaiiGacqWF3oaAdaWgaaWcbaGaemyAaKMaemOAaOMaem4AaSMae8xYdChabeaakiabg2da9maaceaabaqbaeqabiqaaaqaaiabigdaXiabbccaGiabbMgaPjabbAgaMjabbAgaMjabbccaGiabdMgaPjabbccaGiabbggaHjabb6gaUjabbsgaKjabbccaGiabdQgaQjabbccaGiabbEha3jabbwgaLjabbkhaYjabbwgaLjabbccaGiabbwgaLjabbAha2jabbwgaLjabbkhaYjabbccaGiabdUgaRjabbccaGiabb6gaUjabbwgaLjabbggaHjabbkhaYjabbwgaLjabbohaZjabbsha0jabbccaGiabb6gaUjabbwgaLjabbMgaPjabbEgaNjabbIgaOjabbkgaIjabb+gaVjabbkhaYjabbohaZjabbccaGiabbsgaKjabbwha1jabbkhaYjabbMgaPjabb6gaUjabbEgaNjabbccaGiab=L8a3naaBaaaleaacqWGPbqAcqWGQbGAaeqaaaGcbaacbaGae4hmaaJaemiiaaIaee4Ba8MaeeiDaqNaeeiAaGMaeeyzauMaeeOCaiNaee4DaCNaeeyAaKMaee4CamNaeeyzaugaaaGaay5EaaGaeiOla4caaa@8906@

It is 1 if the places of residence of cases *i *and *j *were ever *k*-nearest neighbors during *ω*_*ij*_. Hence *η*_*ijkω *_is 1 if cases *i *and *j *lived near one another at some time when their induction periods overlapped. If their induction periods never overlapped or if they were not *k *nearest neighbors then *η*_*ijkω *_is zero.

### Local test accounting for residential mobility and EIP

Let *N *be the total number of cases. A local statistic for mobile individuals that accounts for the induction period is

Vikω=∑j=1j≠iNηijkω.
 MathType@MTEF@5@5@+=feaafiart1ev1aaatCvAUfKttLearuWrP9MDH5MBPbIqV92AaeXatLxBI9gBaebbnrfifHhDYfgasaacH8akY=wiFfYdH8Gipec8Eeeu0xXdbba9frFj0=OqFfea0dXdd9vqai=hGuQ8kuc9pgc9s8qqaq=dirpe0xb9q8qiLsFr0=vr0=vr0dc8meaabaqaciaacaGaaeqabaqabeGadaaakeaacqWGwbGvdaWgaaWcbaGaemyAaKMaem4AaSgcciGae8xYdChabeaakiabg2da9maaqahabaGae83TdG2aaSbaaSqaaiabdMgaPjabdQgaQjabdUgaRjab=L8a3bqabaaaeaqabeaacqWGQbGAcqGH9aqpcqaIXaqmaeaacqWGQbGAcqGHGjsUcqWGPbqAaaqaaiabd6eaobqdcqGHris5aOGaeiOla4caaa@4780@

We call this the local Vesta statistic after the Roman Goddess of the hearth. It is the count of the *k*-nearest neighbors of case *i *whose induction periods overlapped those of case *i*. This statistic is evaluated about the residential history for each case, and assesses whether and where there is interaction about that case's exposure trace. Its statistical significance is assessed by holding the residential histories constant, and by randomizing the dates of diagnosis with equal probability across the residential histories. The null hypothesis is that an observed date of diagnosis is equiprobable across the *N *cases.

**Figure 2:**
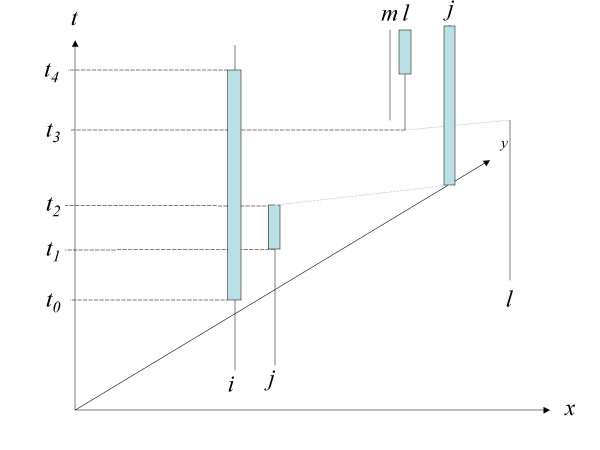
Dynamic topology of residential histories and exposure traces. See text.

It is possible for *V*_*ikω *_to exceed *k*, since the geometry of the residential histories changes through time and *V*_*ikω *_is incremented over case *i*'s exposure trace. To illustrate in Figure 2* x *and *y *indicate geographic space and the vertical axis is time. The residential histories for case *i*, *j*, and *l *are shown as vertical lines. Case *i *never moves and is shown as a continuous, vertical line through time. Exposure traces are shown by long rectangles about a residential history. For example, *ω*_*i *_is indicated by the rectangle about the residential history for subject *i *from *t*_0 _to *t*_4_. Notice case *l *moved place of residence at *t*_3_, and that case *j *moved at *t*_2 _during its induction period *ω*_*j*_. Using *k *= 1 nearest neighbors we see that:

*V*_*i*1*ω *_= 1, since *η*_*ij*1*ω *_= 1 from *t*_1 _to *t*_2 _when *i *and *j *were 1^st ^nearest neighbors.

*V*_*j*1*ω *_= 2, since *η*_*ji*1*ω *_= 1 from *t*_1 _to *t*_2 _when *i *was the 1^st ^nearest neighbor of *j*, and *η*_*jl*1*ω *_= 1 from *t*_3 _to *t*_4 _when *l *was the 1^st ^nearest neighbor of *j*.

*V*_*l*1*ω *_= 0, since case *m*, the first nearest neighbor to *l*, did not have an active exposure trace and *η*_*lm*1*ω *_= 0.

*V*_*m*1*ω *_= 0, since case *m*'s exposure trace never overlapped any others.

### Duration-weighted local interaction statistic

We can extend this to account for the duration of residential stays. Define the duration of time when the induction periods for *i *and *j *overlapped and when *j *was a *k *nearest neighbor of case *i*, and write it as Δ*η*_*ijkω*_. A duration weighted local Vesta is

ΔVikω=∑j=1i≠jNΔηijkω.
 MathType@MTEF@5@5@+=feaafiart1ev1aaatCvAUfKttLearuWrP9MDH5MBPbIqV92AaeXatLxBI9gBaebbnrfifHhDYfgasaacH8akY=wiFfYdH8Gipec8Eeeu0xXdbba9frFj0=OqFfea0dXdd9vqai=hGuQ8kuc9pgc9s8qqaq=dirpe0xb9q8qiLsFr0=vr0=vr0dc8meaabaqaciaacaGaaeqabaqabeGadaaakeaacqqHuoarcqWGwbGvdaWgaaWcbaGaemyAaKMaem4AaSgcciGae8xYdChabeaakiabg2da9maaqahabaGaeuiLdqKae83TdG2aaSbaaSqaaiabdMgaPjabdQgaQjabdUgaRjab=L8a3bqabaaaeaqabeaacqWGQbGAcqGH9aqpcqaIXaqmaeaacqWGPbqAcqGHGjsUcqWGQbGAaaqaaiabd6eaobqdcqGHris5aOGaeiOla4caaa@4A4C@

The units on this statistic are person time (e.g. case days). It quantifies the number of days during case *i's *induction period when its *k*-nearest neighbors were also in their induction periods. Suppose Δ*V*_*i*2*ω *_= 2. This means the induction period for one of its *k *= 2 nearest neighbors was "active" for 2 days during case *i*'s induction period, or that both of it's *k *= 2 nearest neighbors had active induction periods of 1 day during case *i*'s induction period.

### Risk factor and covariate adjustment

We may have knowledge of risk factors and covariates as when a case-control study has been conducted on a subset of the available data. One then can quantify the probability of a given participant being a case, given the risk factors and covariates [22]. Let *p*_*i *_denote the probability of participant *i *being a case given their vector of risk factors and covariates ***x***_*i*_. We would like to construct a version of the local statistic that is sensitive to interaction *above and beyond *that attributable to geographic variation in known risk factors and covariates. We accomplish this by giving decreased weight to those individuals whose cancers are likely attributable to the risk factors and covariates, allowing us to focus our attention on interaction in those cases whose etiology is largely unexplained. For the local Vesta adjusted for covariates

Vikωx=(1−pi)∑j=1i≠jNηijkω(1−pj),
 MathType@MTEF@5@5@+=feaafiart1ev1aaatCvAUfKttLearuWrP9MDH5MBPbIqV92AaeXatLxBI9gBaebbnrfifHhDYfgasaacH8akY=wiFfYdH8Gipec8Eeeu0xXdbba9frFj0=OqFfea0dXdd9vqai=hGuQ8kuc9pgc9s8qqaq=dirpe0xb9q8qiLsFr0=vr0=vr0dc8meaabaqaciaacaGaaeqabaqabeGadaaakeaacqWGwbGvdaWgaaWcbaGaemyAaKMaem4AaSgcciGae8xYdCNaeCiEaGhabeaakiabg2da9iabcIcaOiabigdaXiabgkHiTiabdchaWnaaBaaaleaacqWGPbqAaeqaaOGaeiykaKYaaabCaeaacqWF3oaAdaWgaaWcbaGaemyAaKMaemOAaOMaem4AaSMae8xYdChabeaaaqaabeqaaiabdQgaQjabg2da9iabigdaXaqaaiabdMgaPjabgcMi5kabdQgaQbaabaGaemOta4eaniabggHiLdGccqGGOaakcqaIXaqmcqGHsislcqWGWbaCdaWgaaWcbaGaemOAaOgabeaakiabcMcaPiabcYcaSaaa@560D@

and

ΔVikωx=(1−pi)∑j=1i≠jNΔηijkω(1−pj)
 MathType@MTEF@5@5@+=feaafiart1ev1aaatCvAUfKttLearuWrP9MDH5MBPbIqV92AaeXatLxBI9gBaebbnrfifHhDYfgasaacH8akY=wiFfYdH8Gipec8Eeeu0xXdbba9frFj0=OqFfea0dXdd9vqai=hGuQ8kuc9pgc9s8qqaq=dirpe0xb9q8qiLsFr0=vr0=vr0dc8meaabaqaciaacaGaaeqabaqabeGadaaakeaacqqHuoarcqWGwbGvdaWgaaWcbaGaemyAaKMaem4AaSgcciGae8xYdCNaeCiEaGhabeaakiabg2da9iabcIcaOiabigdaXiabgkHiTiabdchaWnaaBaaaleaacqWGPbqAaeqaaOGaeiykaKYaaabCaeaacqqHuoarcqWF3oaAdaWgaaWcbaGaemyAaKMaemOAaOMaem4AaSMae8xYdChabeaaaqaabeqaaiabdQgaQjabg2da9iabigdaXaqaaiabdMgaPjabgcMi5kabdQgaQbaabaGaemOta4eaniabggHiLdGccqGGOaakcqaIXaqmcqGHsislcqWGWbaCdaWgaaWcbaGaemOAaOgabeaakiabcMcaPaaa@57F9@

for the duration-weighted version. Here *p*_*i *_denotes the probability of participant *i *being a case given their vector of risk factors and covariates ***x***_*i*_. Hence the terms (1 - *p*_*j*_) and (1 - *p*_*i*_) effectively discount the contributions of cases *j *and *i *(respectively) when their cancers reasonably might be attributable by known risk factors and covariates. In practice one will want to calculate the statistics twice, the first time using Equation 4, and the second time adjusting for risk factors and covariates using Equation 6. Comparison of the results identifies cases for which space-time interaction is explained by the risk factors and covariates, and those that are significant both before and after statistical adjustment.

### Global interaction statistics

Equations 3 and 4 quantify local interaction about specific cases. Global tests that assess interaction when all of the cases are considered simultaneously are

Vkω=∑i=1NVikω
 MathType@MTEF@5@5@+=feaafiart1ev1aaatCvAUfKttLearuWrP9MDH5MBPbIqV92AaeXatLxBI9gBaebbnrfifHhDYfgasaacH8akY=wiFfYdH8Gipec8Eeeu0xXdbba9frFj0=OqFfea0dXdd9vqai=hGuQ8kuc9pgc9s8qqaq=dirpe0xb9q8qiLsFr0=vr0=vr0dc8meaabaqaciaacaGaaeqabaqabeGadaaakeaacqWGwbGvdaWgaaWcbaGaem4AaSgcciGae8xYdChabeaakiabg2da9maaqahabaGaemOvay1aaSbaaSqaaiabdMgaPjabdUgaRjab=L8a3bqabaaabaGaemyAaKMaeyypa0JaeGymaedabaGaemOta4eaniabggHiLdaaaa@3EE0@

and

ΔVkω=∑i=1NΔVikω.
 MathType@MTEF@5@5@+=feaafiart1ev1aaatCvAUfKttLearuWrP9MDH5MBPbIqV92AaeXatLxBI9gBaebbnrfifHhDYfgasaacH8akY=wiFfYdH8Gipec8Eeeu0xXdbba9frFj0=OqFfea0dXdd9vqai=hGuQ8kuc9pgc9s8qqaq=dirpe0xb9q8qiLsFr0=vr0=vr0dc8meaabaqaciaacaGaaeqabaqabeGadaaakeaacqqHuoarcqWGwbGvdaWgaaWcbaGaem4AaSgcciGae8xYdChabeaakiabg2da9maaqahabaGaeuiLdqKaemOvay1aaSbaaSqaaiabdMgaPjabdUgaRjab=L8a3bqabaaabaGaemyAaKMaeyypa0JaeGymaedabaGaemOta4eaniabggHiLdGccqGGUaGlaaa@429A@

Equation 7 is an integer count and Equation 8 is duration-weighted. In practice the duration-weighted version is preferred since the duration when exposure traces overlap is of epidemiological interest. When information regarding the probability of being a case is available the global statistics are

Vkωx=∑i=1NVikωx
 MathType@MTEF@5@5@+=feaafiart1ev1aaatCvAUfKttLearuWrP9MDH5MBPbIqV92AaeXatLxBI9gBaebbnrfifHhDYfgasaacH8akY=wiFfYdH8Gipec8Eeeu0xXdbba9frFj0=OqFfea0dXdd9vqai=hGuQ8kuc9pgc9s8qqaq=dirpe0xb9q8qiLsFr0=vr0=vr0dc8meaabaqaciaacaGaaeqabaqabeGadaaakeaacqWGwbGvdaWgaaWcbaGaem4AaSgcciGae8xYdCNaeCiEaGhabeaakiabg2da9maaqahabaGaemOvay1aaSbaaSqaaiabdMgaPjabdUgaRjab=L8a3jabhIha4bqabaaabaGaemyAaKMaeyypa0JaeGymaedabaGaemOta4eaniabggHiLdaaaa@41DA@

and

ΔVkωx=∑i=1NΔVikωx.
 MathType@MTEF@5@5@+=feaafiart1ev1aaatCvAUfKttLearuWrP9MDH5MBPbIqV92AaeXatLxBI9gBaebbnrfifHhDYfgasaacH8akY=wiFfYdH8Gipec8Eeeu0xXdbba9frFj0=OqFfea0dXdd9vqai=hGuQ8kuc9pgc9s8qqaq=dirpe0xb9q8qiLsFr0=vr0=vr0dc8meaabaqaciaacaGaaeqabaqabeGadaaakeaacqqHuoarcqWGwbGvdaWgaaWcbaGaem4AaSgcciGae8xYdCNaeCiEaGhabeaakiabg2da9maaqahabaGaeuiLdqKaemOvay1aaSbaaSqaaiabdMgaPjabdUgaRjab=L8a3jabhIha4bqabaaabaGaemyAaKMaeyypa0JaeGymaedabaGaemOta4eaniabggHiLdGccqGGUaGlaaa@4594@

Here the subscript *kω***x **denote the number of *k *nearest neighbors being considered (*k*), the induction period (*ω*) and the vector of covariates and risk factors **x **for that case.

### Local spatial clustering of exposure traces at time t

Equations 3–6 are accumulated over the exposure traces in the individual life histories. We calculate these local statistics through time, then inspect time plots for shape and inflection points on these monotonically increasing step functions. But because the local Vesta statistics are accumulated over time, they are not particularly sensitive to an ephemeral clustering of exposure traces, since the "signal" added by such clustering is diluted by all that has gone before. We therefore desire a test for local spatial clustering of exposure traces at any given time *t*. We would like this statistic to tell us, when considering case *i*, whether its *k*-nearest neighbors tend to have "active" exposure traces. Define

cit={IFF case i is in its exposure trace at time t (t∈ωi)0 otherwise
 MathType@MTEF@5@5@+=feaafiart1ev1aaatCvAUfKttLearuWrP9MDH5MBPbIqV92AaeXatLxBI9gBaebbnrfifHhDYfgasaacH8akY=wiFfYdH8Gipec8Eeeu0xXdbba9frFj0=OqFfea0dXdd9vqai=hGuQ8kuc9pgc9s8qqaq=dirpe0xb9q8qiLsFr0=vr0=vr0dc8meaabaqaciaacaGaaeqabaqabeGadaaakeaacqWGJbWydaWgaaWcbaGaemyAaKMaemiDaqhabeaakiabg2da9maaceaabaqbaeqabiqaaaqaaiabbMeajjabbAeagjabbAeagjabbccaGiabbogaJjabbggaHjabbohaZjabbwgaLjabbccaGiabdMgaPjabbccaGiabbMgaPjabbohaZjabbccaGiabbMgaPjabb6gaUjabbccaGiabbMgaPjabbsha0jabbohaZjabbccaGiabbwgaLjabbIha4jabbchaWjabb+gaVjabbohaZjabbwha1jabbkhaYjabbwgaLjabbccaGiabbsha0jabbkhaYjabbggaHjabbogaJjabbwgaLjabbccaGiabbggaHjabbsha0jabbccaGiabbsha0jabbMgaPjabb2gaTjabbwgaLjabbccaGiabdsha0jabbccaGiabbIcaOiabdsha0jabgIGioJGaciab=L8a3naaBaaaleaacqWGPbqAaeqaaOGaeiykaKcabaGaeGimaaJaeeiiaaIaee4Ba8MaeeiDaqNaeeiAaGMaeeyzauMaeeOCaiNaee4DaCNaeeyAaKMaee4CamNaeeyzaugaaaGaay5Eaaaaaa@8121@

The spatial clustering test is then

Sikωt=cit∑j=1kcjt
 MathType@MTEF@5@5@+=feaafiart1ev1aaatCvAUfKttLearuWrP9MDH5MBPbIqV92AaeXatLxBI9gBaebbnrfifHhDYfgasaacH8akY=wiFfYdH8Gipec8Eeeu0xXdbba9frFj0=OqFfea0dXdd9vqai=hGuQ8kuc9pgc9s8qqaq=dirpe0xb9q8qiLsFr0=vr0=vr0dc8meaabaqaciaacaGaaeqabaqabeGadaaakeaacqWGtbWudaWgaaWcbaGaemyAaKMaem4AaSgcciGae8xYdCNaemiDaqhabeaakiabg2da9iabdogaJnaaBaaaleaacqWGPbqAcqWG0baDaeqaaOWaaabCaeaacqWGJbWydaWgaaWcbaGaemOAaOMaemiDaqhabeaaaeaacqWGQbGAcqGH9aqpcqaIXaqmaeaacqWGRbWAa0GaeyyeIuoaaaa@4499@

The summation is over case *i*'s *k *nearest neighbors. We call this the Janus statistic, after the Roman God who guarded the doorway to the home. Janus is the count, at time *t*, of the number of *k *nearest neighbors of case *i *with overlapping induction periods. Notice the statistic can be non-zero only when case *i *is in its induction period. If we define the time interval Δ*t *such that the geography of the residential histories doesn't change (e.g. none of the cases moves place of residence, and whether case *i *and its neighbors are in their respective induction periods doesn't change) we may consider the time weighted version of the statistic

ΔSikωt=Δt cit∑j=1kcjt
 MathType@MTEF@5@5@+=feaafiart1ev1aaatCvAUfKttLearuWrP9MDH5MBPbIqV92AaeXatLxBI9gBaebbnrfifHhDYfgasaacH8akY=wiFfYdH8Gipec8Eeeu0xXdbba9frFj0=OqFfea0dXdd9vqai=hGuQ8kuc9pgc9s8qqaq=dirpe0xb9q8qiLsFr0=vr0=vr0dc8meaabaqaciaacaGaaeqabaqabeGadaaakeaacqqHuoarcqWGtbWudaWgaaWcbaGaemyAaKMaem4AaSgcciGae8xYdCNaemiDaqhabeaakiabg2da9iabfs5aejabdsha0jabbccaGiabdogaJnaaBaaaleaacqWGPbqAcqWG0baDaeqaaOWaaabCaeaacqWGJbWydaWgaaWcbaGaemOAaOMaemiDaqhabeaaaeaacqWGQbGAcqGH9aqpcqaIXaqmaeaacqWGRbWAa0GaeyyeIuoaaaa@499D@

This statistic is measured in case-time units, e.g. case-days.

### Focused spatial clustering of exposure traces at time t

Suppose we know the address history of a putative source of a carcinogen, such as an industry. Given focus *f *we denote this address history as ***F***_*f*_. Further suppose we have information regarding the emission volume per unit time, such as might come from EPA's TRI (Toxic Release Inventory) data. Call this ***E***_*f*_(*t*). The *i*^*th *^case has induction period *ω*_*i *_that begins at *t*_*i*0 _and ends at *t*_*i*1_. An emission-weighted focused Vesta statistic is then

ΔVfikω=∑i=1k∫ti0ti1Ef(t)dt.
MathType@MTEF@5@5@+=feaafiart1ev1aaatCvAUfKttLearuWrP9MDH5MBPbIqV92AaeXatLxBI9gBaebbnrfifHhDYfgasaacH8akY=wiFfYdH8Gipec8Eeeu0xXdbba9frFj0=OqFfea0dXdd9vqai=hGuQ8kuc9pgc9s8qqaq=dirpe0xb9q8qiLsFr0=vr0=vr0dc8meaabaqaciaacaGaaeqabaqabeGadaaakeaacqqHuoarcqWGwbGvdaWgaaWcbaGaemOzayMaemyAaKMaem4AaSgcciGae8xYdChabeaakiabg2da9maaqahabaWaa8qCaeaacqWGfbqrdaWgaaWcbaGaemOzaygabeaakiabcIcaOiabdsha0jabcMcaPiabdsgaKjabdsha0bWcbaGaemiDaq3aaSbaaWqaaiabdMgaPjabicdaWaqabaaaleaacqWG0baDdaWgaaadbaGaemyAaKMaeGymaedabeaaa0Gaey4kIipaaSqaaiabdMgaPjabg2da9iabigdaXaqaaiabdUgaRbqdcqGHris5aOGaeiOla4caaa@510D@

Here the summation is over the cases that are *k *nearest neighbors of focus *f*. This statistic will be large when the emission volume of the focus tends to be elevated during times that coincide with the induction periods of its *k*-nearest neighbors.

### Sensitivity of interaction statistics to specification of the EIP

At least two instances may arise regarding specification of *ω *and *τ*. The first arises when we are able to model *ω *and *τ *as a function of individual-level characteristics such as genetics, life course, covariates and risk factors. The second arises when we have little knowledge of how *ω *and *τ *may vary from one individual to another. One then may specify *ω *under the simplifying assumption that *ω*_1 _= *ω*_2 _= ... = *ω*_*N*_. The remainder of this paper deals with the second instance, since it is more generally applicable in the absence of the ability to directly observe *ω *and *τ*, and since models of induction period as a function of genetics, risk factors and covariates are typically not available. Given a model of EIP, we follow these steps to assess sensitivity of the interaction statistics.

1. Define the model of EIP and the values of the parameters to explore.

a. Example: For the bladder cancer study we will explore 110 combinations of the induction (1,3,5,7,9,11,13,15,17,19) and latency (5,7,9,11,13,15,17,19,21,23,25) periods.

2. For each parameter set evaluate the distribution of the test statistics under the null hypothesis.

a. Under the null hypothesis of no association between residential history and age at diagnosis allocate the ages at diagnosis with equal probability across the residential histories, calculating the tests for interaction each time. This step is repeated 999 times to generate the distribution of the test statistic under the null hypothesis. For Janus one uses a conditional randomization that keeps the date of diagnosis for the case being considered the same (not randomized). For the Janus statistic, which is a local test, the randomization is conditional in the sense that the date of diagnosis for the case being considered is held constant to be the observed date of diagnosis for that case. The dates of diagnosis for the remaining cases are randomized.

b. Compare the value of the test statistic for the original data to the distribution of the test statistic under the null hypothesis from step 2a. A p-value for a given statistic is calculated for each parameter set.

3. One then inspects the p-values of the global Vesta to identify induction and latency periods that result in significant global interaction. The local statistics may then be used to identify those locations and times contributing the most to the significant global interaction.

### The diagnostic process

**Figure 3:**
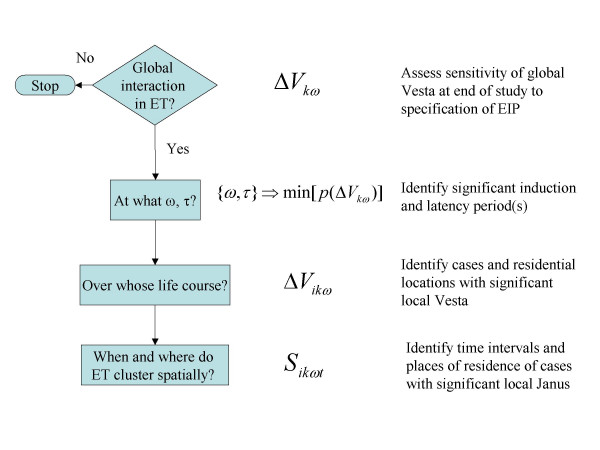
Diagnostic process for exposure traces, see text.

A diagnostic process identifies those induction periods and latencies that maximize clustering in exposure traces, while also ameliorating multiple testing (Figure 3). We first use the probability of the global Vesta to assess whether a given latency and induction period is significant (Figure 3, "Global interaction in exposure traces?"). This step is repeated for all sets of induction and latency periods being considered. If none are significant, we advocate for the analysis to cease. While local clustering may be significant [23], as a strategy for ameliorating multiple testing, we only advise searching for those local clusters if the signal is strong enough to also produce a significant global cluster statistic. Those global Vesta statistics (if any) that result in significant global interaction are retained (Figure 3, "At what *ω*, *τ*?"), and used to identify the cases, residential locations and times when significant local interaction occurred (Figure 3, "Over whose life course?"). Finally, Janus is applied to identify the locations and times of significant spatial clustering in exposure traces (Figure 3, "When and where do ET cluster spatially?").

### The bladder cancer data set

A population-based bladder cancer case-control study is underway in southeastern Michigan and was used in both simulated and applied studies. Cases diagnosed in the years 2000–2004 and living in Genesee, Huron, Ingham, Jackson, Lapeer, Livingston, Oakland, Sanilac, Shiawassee, Tuscola, and Washtenaw counties are being recruited from the Michigan State Cancer Registry. Controls from this study are used by us to quantify the probability of being a case given risk factors and covariates. Controls are being frequency matched to cases by age (± 5 years), race, and gender, and are being recruited using a random digit dialing procedure from an age-weighted list. At this stage of recruitment, controls are not adequately matched; therefore, age, race, and gender are adjusted for in the analyses. To be eligible for inclusion in the study, participants must have lived in the eleven county study area for at least the past 5 years and have had no prior history of cancer (with the exception of non-melanoma skin cancer). Participants are offered a modest financial incentive and research is approved by the University of Michigan IRB-Health Committee. The data analyzed here are from 374 cases and 490 controls. Refer to [24] for details on geocoding residential histories.

### The simulation study design

To evaluate type I and type II error we undertook simulations using the residential histories of the cases from the bladder cancer study, but assigned new times of diagnosis based on different scenarios for which the modeled degree of interaction was under experimental control. In each of our experiments we explored sensitivity of the results to pair-wise combinations of induction (1, 3, 5, 7 and 9 years) and latency (5, 7, 9, 11, 13, 15, 17 and 19 years). Three scenarios were analyzed using *k *= 1 and *k *= 5 nearest neighbors.

#### 1) No interaction

This scenario explored the type I error of the global statistic and the sensitivity of the type I error to specification of induction period and latency. We arbitrarily assigned each case a new date of diagnosis drawn from a uniform distribution between 1990 and 2005, resulting in a dataset without space-time interaction. We then plotted the probability of the global Vesta as a function of the induction and latency periods. This allowed us to evaluate the sensitivity of the global statistic to specification of these parameters when the null hypothesis was true.

#### 2) Cluster of Size 10

We modeled a local exposure in early 1985 that resulted in cancers in the exposed group with an induction period of 1 year and a latency of 15 years, resulting in peak years of diagnosis in 1999–2000. We swapped the diagnosis dates for the exposed group with randomly selected members of the remaining cases whose dates of diagnosis were in 1999–2000. This maintained the distribution of dates of diagnosis, and corresponds to an ephemeral exposure of brief duration.

#### 3) Cluster of size 25

**Figure 4:**
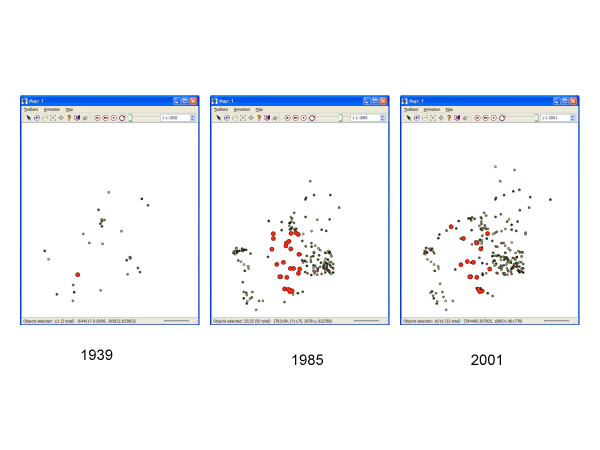
Evolution of the cluster of size 25. Locations of place of residence of cluster members are shown as red circles in 1939 (left), during the exposure in 1985 (center) and in 2001 (right).

We modeled a cluster of size 25 occurring in 1985 and incorporating members of cluster size 10 (Figure 4). The induction period (1 year) and latency period (15 years) were maintained.

### Analysis of bladder cancer in Michigan

Once we had obtained a clearer understanding of the statistical performance and sensitivity of the new methods we applied them to the cases from the bladder cancer study using the original dates of diagnosis. We evaluated *k *= 1 and *k *= 5, but increased the range of the parameters considered for the induction and latency periods. We plotted the probability of the global statistic as a function of the EIP, and for that induction and latency period that resulted in significant global interaction inspected maps of the local statistics to identify clusters of high space-time interaction through time. We then adjusted the tests for known risk factors (smoking) and covariates using the methods described in equation 6. Comparison of the graphs of the probability of the global Vesta as a function of EIP and maps for the tests before and after adjustment allowed us to identify (1) possible contributions of the risk factors and covariates to the induction and latency periods and (2) those local clusters that cannot be explained by smoking and covariates. Clusters that cannot be explained by known factors are of particular interest, as they may be caused by exposures that were not assessed in the case-control study.

## Results

### Simulation study

#### No Interaction

The plot of the probability of the global Vesta as a function of the parameter values has a minima at 0.107 and a maxima near 1. At an alpha level of 0.05, one would correctly conclude there was no space-time interaction.

We then calculated the values of each of the local statistics through time, and evaluated their significance at each unique arrangement of places of residence. This allowed us to construct graphs of the observed proportion of local statistics that were correctly classified as "not clustered" as a function of the decision criteria for the test. We inspected curves for each parameter set. The correct decision of no interaction is achieved 100% of the time up to a decision level for the test of over 30%. For the scenario considered, the risk of false positives is zero and does not increase until the alpha level of the test is above 0.3.

#### Cluster Size 10

We applied the global Vesta from Equation 8, repeating the analysis for each of the 40 parameter sets. We then plotted its probability as a function of the EIP. A minimum p-value of 0.034 was observed at an EIP of 16 years, corresponding to induction period 1 year and latency of 15 years, the same induction and latency used when modeling the cluster.

We next used the local Vesta to identify those cases experiencing significant interaction over their life course, and the local Janus statistic to find those times when exposure traces clustered. Even though the modeled cluster was ephemeral and small (10 cases), the Vesta and Janus statistics correctly identified its timing, the induction and latency periods used, and found 5 of the cases in the modeled cluster.

#### Cluster Size 25

The sensitivity analysis to specification of EIP found minimum p < 0.01 for the global statistic for an average induction period of 2.7 years and an average latency of 14.7 years, near that of the modeled cluster. The Janus statistic correctly localized the cluster in time, and identified 21 members of the cluster, with 4 false negatives and no false positives. The approach thus appears capable of estimating with acceptable accuracy the latency, induction periods and membership of the simulated clusters.

### Bladder cancer

**Figure 5:**
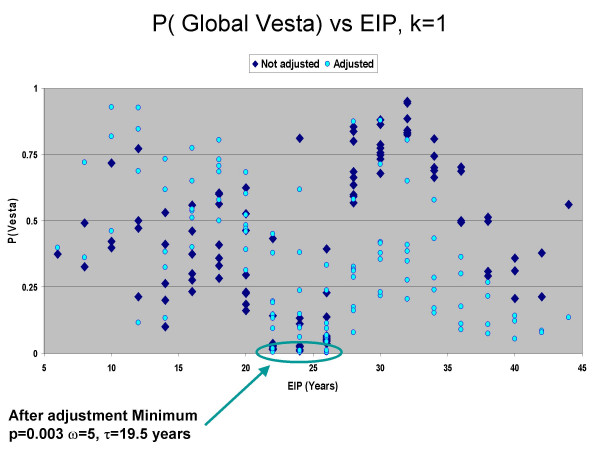
Empirical Induction Period sensitivity analysis, bladder cancer study, *k *= 1. The probability of the global statistic for space-time interaction is on the *y*-axis, the *x*-axis is the EIP in years used when evaluating the global statistic. A minimum of p = 0.003 is reached at an average induction period of 5 years, and a latency of 19.5 years.

**Figure 6:**
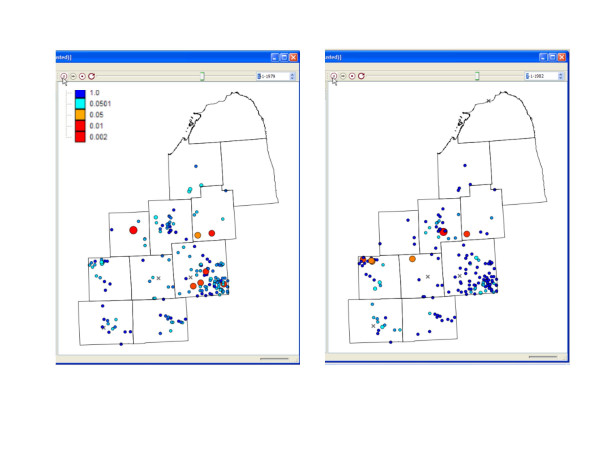
Local spatial clustering of exposure traces for bladder cancer cases. Shown are the locations of significant clusters for the Janus statistic on 1/1/1979 (left) and 7/1/1982 (right).

We next analyzed the bladder cancer data to better understand how this new approach might be applied to real data. We analyzed a total of 110 parameter sets using induction periods 1, 3, 5, 7, 9, 11, 13, 15, 17, 19 and latencies 5, 7, 9, 11, 13, 15, 17, 19, 21, 23 and 25 years. This resulted in EIP's from 6 to 44 years. We employed logistic regression and the case and control data to quantify the probability of being a case given the risk factor smoking and the covariates age, gender, education and race (for further description of the logistic model see [22]). We then ran the analyses taking into account these case probabilities, employing the method of Equation 6, and undertook the same analyses without covariate adjustment. We evaluated *k *= 1 and *k *= 5 to explore scale dependencies in case clustering. The results using *k *= 5 were not statistically significant, but were for *k *= 1. After adjustment, the smallest probabilities of the global Vesta were for EIP's from 22 to 26 years (Figure 5), with a minima of p = 0.003 occurring at average induction period 5 years, latency 19.5 years. We used these as input to Janus to evaluate local spatial clustering of exposure traces through time. Significant clustering of exposure traces begins in 1975 and continues through 1986 (Figure 6).

## Discussion

The effects of latency as described in current epidemiological literature are often insufficient to address public health questions, largely because quantitative models of latency are lacking [24]. Langholz et al. [24] developed latency models based on bilinear and exponential decay functions, and fitted these models to case-control data within a likelihood framework. They defined latency as the function describing how the relative risk associated with a *known exposure *changes through time, and the function may be estimable in occupational studies. As an example, they observed that "... relative risk associated with exposure increases for about 8.5 years and thereafter decreases until it reaches background levels after about 34 years" in a study of lung cancer in a cohort of uranium miners. In contrast, Janus and Vesta evaluate whether the residential histories of cases exhibit interaction during the induction periods – those times when causative exposures plausibly might have occurred – but *we do not necessarily know what those exposures might be*. We thus must use our admittedly inadequate knowledge of cancer latency to define induction periods within which an environmental exposure *might *be causally associated with a given case. This could indicate, for example, those times in a person's life course when exposures (should they occur) are most likely to cause cancer. Several authors have suggested, that when faced with uncertainty, one should explore sensitivity of the latency-based statistic to plausible specifications of the induction period [21,25], and that is the approach used in this paper.

The Janus statistic is sensitive to ephemeral spatial clustering of exposure traces, and the simulation studies found that it can pick up the signal from a cluster of brief duration. The Vesta statistics are accumulated over the induction periods, and identify cases who were in close geographic proximity to other cases during their induction periods. The global Vesta thus evaluates interaction in exposure traces at specific induction and latency periods. When interaction is absent the simulations found the global Vesta not significant even when a large number of values of the induction and latency periods are considered. Hence adjustment for multiple testing may not be required to correct the type I error when evaluating a range of empirical induction periods, provided one uses the diagnostic process and first evaluates whether the global statistics are significant before proceeding. Additional simulation studies are needed to evaluate whether this holds over a range of scenarios.

As noted earlier, the simulations we conducted are limited, and it may very well be that false positives will arise under other simulated conditions. Given the simulations we have conducted to date, one possible explanation is that the methods are more prone to type II error than they are to type I error. This kind of a trade off between type I and type II error is observed for many statistical methods. Further simulation studies are needed to more fully explore the trade offs between type I error, type II error, and statistical power.

Statistical significance of the global Vesta is used to determine (1) whether the analysis should proceed, and (2) what induction and latency periods to employ for the local analyses. The diagnostic framework thus is designed to detect "big signals" that will result in statistical significance of the global Vesta. We do not employ corrections for multiple testing of the local Vesta once significance of the global Vesta has been demonstrated; rather we seek to identify those cases and time periods that contribute the most to a significant global test statistic. The validity of this approach is supported by simulation, in which clusters of size 25 and even of size 10 were localized with small type I error, and returned appropriate induction and latency periods. Janus found 5 members of the cluster of size 10 and 21 members of the cluster of size 25, with cases that were missed occurring on the cluster edge. This seems to be reasonable performance given the small cluster size and the ephemeral nature of the modeled clusters.

When considering multiple testing, Fuchs and Kenett [23] argued, in the aspatial case, that a test of the most extreme local statistic (accounting for multiple testing) can be more powerful at finding clusters than the use of the corresponding global test. This likely may be true for spatial tests as well, in which case significant local clusters might be identified even when the global statistic is not significant.

Several caveats apply to the simulation design. We constructed the simulations to be simple, and yet to pose a fairly stringent "first test" of the new methods by modeling clusters of short duration and size. We decided to swap dates of diagnosis when constructing the clusters, making interaction and clustering of exposure traces the only aspect of the dataset that would change across simulations – the frequency distribution of dates of diagnosis was constant. We used a cluster of size 10 and 1 year duration as the smallest, and were pleasantly surprised to find the methods indeed were sensitive enough to find that cluster. Nonetheless, additional simulations are needed to address the impacts of uncertainty in the residential histories, multiple clusters, and of heterogeneity in individual induction and latency periods.

In order to generate bias in interaction of the exposure traces one would need to preferentially sample a subset of the population with similar dates of diagnosis that were in geographic proximity to one another during their induction periods. This might occur for rural populations characterized by little residential mobility. At first blush a second potential source of bias might be differential mobility in different parts of the study area. Localities with greater residential mobility might have larger variability in the temporal overlap of exposure traces, since individuals on average do not stay as long in any given place of residence. The randomization procedure holds the residential histories as a given, and permutes dates of diagnosis across the cases. Differential residential mobility should therefore be accounted for under the null hypothesis. Finally, changes in diagnostic procedures such that risk of diagnosis increases at different times in different parts of the study area are a potential source of bias, since this would lead to an apparent overlap in exposure traces. This would definitely create clustering at time of diagnosis, but we'd expect the cluster to become diffuse by time of the induction period due to residential mobility, unless the induction period is close to time of diagnosis.

At the time this article was written the bladder cancer study was in progress and cases were still being enrolled. A portion of the thumb of Michigan – those counties in the North of the study area – have yet to be visited by the field teams for the latest round of sampling. These comprise a primarily rural population with recent dates of diagnosis, a potential source of sampling bias (i.e., differential sampling across the study area) that could result in spurious findings of significant interaction. We thus must wait before attaching further interpretation to clusters of exposure traces found under the Janus and local Vesta statistics.

What is the reason for this differential sampling? For the bladder cancer study differential sampling arose because of the timeline chosen for household visits to residences of the cases and controls. These visits included survey instruments, water sampling to assess arsenic concentrations in the water supply, and biological sampling such as toenail clippings and bucal samples to assess recent arsenic exposure and genetic factors. Many of these sample instruments and assays were tangential to the topic of the current paper, and are discussed in detail in other peer-reviewed publications. Differential sampling at the time of this writing arose because sampling is systematic geographically in order to reduce expense – the sampling team goes into an area (say the southern part of the study area) and visits those residences, at a later date visits residences in another area, and so on. Hence while the overall sample is representative, the manner in which the data are collected is geographically and temporally sequential. Thus when we analyze data before data collection is complete our sample up to that point in time necessarily is differential. This of course will not be an issue when we conduct analyses after data collection is finished.

If these clusters persist once data collection is complete, we will need to investigate environmental agents hypothesized to cause bladder cancer that produce an induction period of five years, followed by a latency period of nearly twenty years. In addition, the agent or agents responsible only resulted in clusters using one nearest neighbor, not the nearest five neighbors, suggesting tight geographic areas of high exposure. One might conjecture that a possible cause of this space-time clustering pattern is pollution from several local industries in the region [12], or a more disperse contaminant that appears in localized hotspots, such as arsenic in private well water which is found in elevated concentrations in southeastern Michigan [26]. Examination of these hypotheses will involve thorough exposure assessment; however, the space-time clustering approach introduced here can help bring these possible exposures to light. These analyses will be repeated once data collection is complete.

The strength of the Janus and Vesta statistics lies in their ability to help identify induction and latency periods, an area of research generally underserved in cancer epidemiology. Most efforts aimed at understanding the temporal relationship between exposure and cancer have focused on improving the temporal resolution of exposure assessments [26-28]. In this paper, we take advantage of disease and residential history datasets for gaining insights about the temporal dynamics of the exposure-disease relationship. We developed statistics for quantifying space-time interaction in exposure traces, while allowing the user to explore a range of induction and latency periods. If clustering is identified after following this approach, this calls for investigation into temporally characterized exposures potentially responsible for the clustering.

These new methods raise the possibility of routine surveillance using cancer registry data in a manner that accounts for individual mobility, identifies plausible values of the induction and latency periods, and that identifies geographic locations and times associated with increases in cancer risk *above and beyond *that expected given known covariates and risk factors in geographically mobile populations.

## Authors' contributions

GJ created the Vesta and Janus statistics, undertook the simulation experiments and bladder cancer analyses, and drafted the ms. JM identified the need for interaction statistics for mobile populations, undertook the regression analyses for specifying the case probabilities, and coordinated preparation of the bladder cancer data. AK programmed the methods in the Space-Time Intelligence System. All authors read and approved the final manuscript.
